# Accuracy of a Fourth-Generation Subcutaneous Continuous Glucose Sensor

**DOI:** 10.1089/dia.2017.0087

**Published:** 2017-08-01

**Authors:** Mark P. Christiansen, Satish K. Garg, Ronald Brazg, Bruce W. Bode, Timothy S. Bailey, Robert H. Slover, Ashley Sullivan, Suiying Huang, John Shin, Scott W. Lee, Francine R. Kaufman

**Affiliations:** ^1^ Diablo Clinical Research, Walnut Creek, California.; ^2^ Barbara Davis Center for Diabetes, Aurora, Colorado.; ^3^ Rainier Clinical Research Center, Renton, Washington.; ^4^ Atlanta Diabetes Associates, Atlanta, Georgia.; ^5^ AMCR Institute, Escondido, California.; ^6^ Medtronic, Northridge, California.

**Keywords:** Glucose sensor, Sensor-integrated pump, Sensor accuracy, Mobile device, MARD

## Abstract

***Background:*** This study evaluated the accuracy and performance of a fourth-generation subcutaneous glucose sensor (Guardian^™^ Sensor 3) in the abdomen and arm.

***Methods:*** Eighty-eight subjects (14–75 years of age, mean ± standard deviation [SD] of 42.0 ± 19.1 years) with type 1 or type 2 diabetes participated in the study. Subjects wore two sensors in the abdomen that were paired with either a MiniMed^™^ 640G insulin pump, or an iPhone^®^ or iPod^®^ touch^®^ running a glucose monitoring mobile application (Guardian Connect system) and a third sensor in the arm, which was connected to a glucose sensor recorder (GSR). Subjects were also asked to undergo in-clinic visits of 12–14 h on study days 1, 3, and 7 for frequent blood glucose sample testing using a Yellow Springs Instrument (YSI) reference.

***Results:*** The overall mean absolute relative difference (MARD ± SD) between abdomen sensor glucose (SG) and YSI reference values was 9.6% ± 9.0% and 9.4% ± 9.8% for the MiniMed 640G insulin pump and Guardian Connect system, respectively; and 8.7% ± 8.0% between arm SG and YSI reference values. The percentage of SG values within 20% agreement of the YSI reference value (for YSI >80 mg/dL) was 90.7% with the MiniMed 640G insulin pump, 91.8% with the Guardian Connect system, and 93.1% for GSR-connected arm sensors. Mean functional sensor life, when calibrating 3–4 times/day, was 145.9 ± 39.3 h for sensors paired with the MiniMed 640G insulin pump, 146.1 ± 41.6 h for sensors paired with the Guardian Connect system, and 147.6 ± 40.4 h for sensors connected to the GSR. Responses to survey questions regarding sensor comfort and ease of use were favorable.

***Conclusions:*** The Guardian Sensor 3 glucose sensor, whether located in abdomen or the arm, provided accurate glucose readings when compared with the YSI reference and demonstrated functional life commensurate with the intended 7-day use.

****ClinicalTrials.gov**:** NCT02246582

## Introduction

Continuous glucose monitoring (CGM), relative to intermittent self-monitored blood glucose (SMBG) values, has afforded significant improvements in the management of diabetes mellitus. The ability to visualize current and trending interstitial glucose values through real-time CGM (RT-CGM) with multiple daily injections therapy has demonstrated reductions in glycated hemoglobin (HbA_1c_) levels or different glucose variability indexes.^1–4^ Findings from sensor-augmented and sensor-integrated pump studies have demonstrated not only improved HbA_1c_ levels^5–7^ and reduced glucose variability,^4,7,8^ but also reduced hypoglycemia,^9,10^ improved treatment satisfaction,^11,12^ and projected improvements in cost effectiveness.^13^ To add, the consistency of sensor wear or CGM system use has also been shown to link directly with therapy effectiveness.^7,14,15^

At the core of CGM is the accuracy at which the subcutaneous sensor performs. Previous prospective, multicenter studies investigating earlier sensors of intended 6-day use demonstrated overall mean absolute relative difference (MARD) values of 13.9%^16^ and 13.6%.^17^ In contrast to the prior glucose sensors, the recently developed Guardian Sensor 3 sensor (Medtronic, Northridge, CA) has several enhancements: a 7-day functional life and an updated chemistry stack and optimized electrode design to improve in vivo stability and performance. The updated algorithm in the new sensor's transmitter has a more rapid and adaptive response to calibrations that enhances accuracy; improved signal processing that reduces sensor signal noise while minimizing delay; and the ability to reduce outliers through advanced diagnostics and fault detection. This sensor was developed to advance Medtronic CGM products to a level suitable for use in a hybrid closed-loop (HCL) system. The performance of the Guardian Sensor 3 glucose sensor when inserted in the abdomen and arm, and communicating with an insulin pump, mobile device application, or glucose sensor recorder (GSR) are reported herein. The safety and glucose profiles of the sensor, as part of the MiniMed 670G HCL system, have been previously reported.^18,19^

## Methods

This prospective randomized study was conducted at six investigational centers in the United States and enrolled 93 subjects (14–75 years of age) diagnosed with type 1 or type 2 diabetes with a duration of ≥12 months. Additional inclusion criteria for study participation included adequate venous access, as assessed by the investigational team, and established insulin-carbohydrate and insulin-sensitivity ratios for subjects selected to participate in the hyperglycemic and hypoglycemic challenges. Exclusion criteria included hypoglycemic seizure, loss of consciousness, or an episode of diabetic ketoacidosis (DKA) within 6 months of the screening visit; a history of central nervous system or seizure disorder; cardiac disorder resulting in syncope; myocardial infarction, unstable angina, coronary artery bypass surgery, coronary artery stenting, transient ischemic attack, cerebrovascular accident, angina, congestive heart failure, ventricular rhythm disturbances, or thromboembolic disease; a hematocrit lower than the normal reference range; a history of adrenal insufficiency; the inability to tolerate tape adhesive in the area of sensor placement; any unresolved adverse skin condition in the area of sensor or device placement (e.g., psoriasis, rash, *Staphylococcus* infection); or active participation in an investigational study (drug or device) where treatment was received within 2 weeks of the screening visit. Additional exclusion criteria for female subjects included positive pregnancy test, unwillingness to use a form of contraception deemed reliable by the investigator, or a pregnancy planned during the course of the study. A central laboratory certified by the National Glycohemoglobin Standardization Program methodology was used to determine baseline HbA_1c_ values. However, subjects were not excluded based on baseline HbA_1c_ values. The study was approved by respective institutional review boards and informed consent was obtained from all subjects at each investigational site.

### Study design

**FIG. 1.**
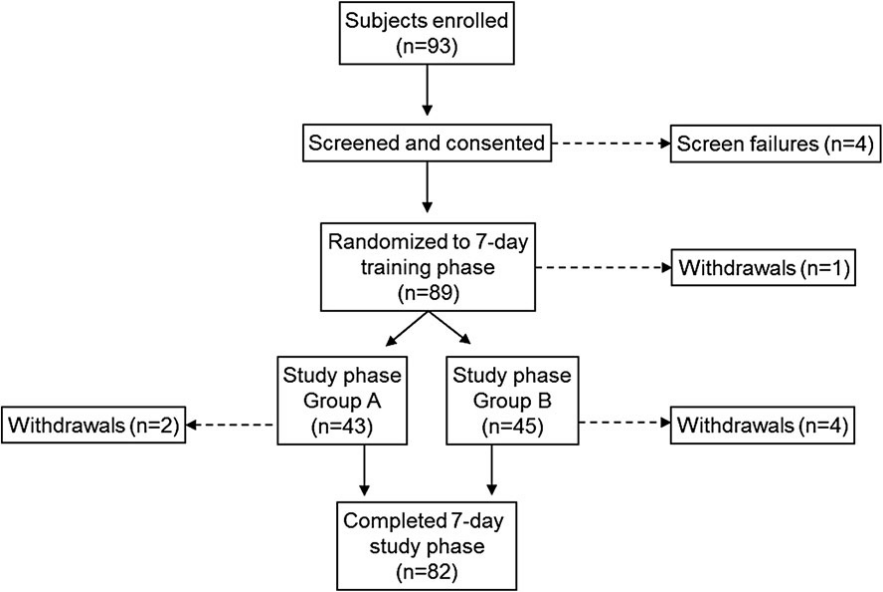
Study flow and subject disposition.

There were 93 subjects enrolled; 88 completed the 7-day training phase, and 82 completed the 7-day study phase (Fig. 1). There were four screen failures before randomization into the training phase and one subject withdrew during the training phase. The reasons for six subjects not completing the study phase included withdrawal due to competing school, work, or other schedules (*n* = 5) and a missed sensor-insertion visit (*n* = 1).

During the training phase, subjects were trained in sensor insertion and removal, as well as use of the other study devices and procedures. Subjects who met the inclusion/exclusion criteria were randomized 1:1 to Group A or Group B. Group A subjects had day 1 frequent sample testing (FST) performed immediately following sensor insertion, and day 3 and day 7 testing performed ∼50 and 146 h after sensor insertion. Subjects assigned to Group B underwent day 1 FST ∼14 h after sensor insertion, and day 3 and day 7 testing performed 62 and 158 h after sensor insertion. Randomization of subjects to Group A or Group B provided sensor data across the full 24 h of day 1, day 3, and day 7 of sensor life. No comparison of results between Group A and Group B subjects was performed. Subjects were provided study devices that included the MiniMed 640G insulin pump (Medtronic), the Guardian Connect system application (Medtronic) installed on an iPhone or iPod touch, a Glucose Sensor Recorder (GSR, Medtronic), Guardian Sensor 3 sensor (Medtronic), Guardian Link 3 transmitter (Medtronic) compatible with the MiniMed 640G insulin pump, Guardian Connect transmitter (Medtronic) communicating with the Guardian Connect system application, a CONTOUR^®^NEXT Link blood glucose meter and CONTOURNEXT Blood Glucose Test Strips (Ascensia Diabetes Care, Parsippany, New Jersey).

During the study phase, each subject wore three sensors. One sensor, inserted in the abdominal area, was connected to the Guardian Link 3 transmitter, which was paired with the MiniMed 640G insulin pump. The insulin pump was not used to deliver insulin or manage diabetes. The second sensor, also inserted in the abdominal area, was connected to the Guardian Connect transmitter paired with the iPhone or iPod running the Guardian Connect system application. The third sensor, inserted in the upper arm area, was connected to a GSR.

Subjects were asked to undergo three in-clinic visits for YSI (Yellow Springs Instrument) FST periods, lasting 12–14 h, where intravenous (IV) blood samples were drawn every 5–15 min and analyzed using the 2300 STAT Plus™ Glucose & Lactate Analyzer (YSI Life Sciences, Yellow Springs, OH). During FSTs, only the subjects with established insulin sensitivity and insulin–carbohydrate ratios underwent hypoglycemic and hyperglycemic challenges. During the hypoglycemic challenge, glucose levels were lowered (under supervision) to a target of 50–75 mg/dL for ∼1 h, including 15 min between 50 and 60 mg/dL. During the hyperglycemic challenge, higher glucose levels were achieved by standard meal administration to a target of 180–400 mg/dL for ∼2 h, including 30 min between 350 and 400 mg/dL if possible, as allowed by the provider. A subcohort of subjects, 10 from each group, were asked to exercise for, at least, 30 min during YSI FST and, at least, two times for a minimum of 20 min per day between the first and last YSI FSTs.

Throughout the study, subjects were instructed to manage their diabetes independent of the sensor glucose (SG) information displayed by the MiniMed 640G insulin pump or the Guardian Connect system application on the iPhone or iPod. During the home use (outside of clinic) portion of the study, subjects were instructed to calibrate the sensors paired with the MiniMed 640G insulin pump and the Guardian Connect system four times daily or when prompted by a calibration alert. During the FST, sensors were calibrated based on prompts from the MiniMed 640G insulin pump or Guardian Connect system. Calibrations were required 40–120 min after sensor insertion, 6 h after the first calibration, 12 h after the first calibration, and every 12 h, thereafter. Additional calibrations may have been requested by the MiniMed 640G insulin pump or Guardian Connect system, according to sensor signal integrity detected by the transmitter algorithm. The raw data stored in the GSR were processed after data collection using the calibration values prospectively entered in the MiniMed 640G insulin pump and the same algorithm used by the Guardian Link 3 transmitter. This allowed modeling of the SG values that would have been presented in real-time, if the sensor placed in the arm had been connected to a Guardian Link 3 transmitter instead of the GSR.

Sensor accuracy was evaluated by comparing YSI reference plasma glucose values, obtained during FST, to SG values obtained by the MiniMed insulin 640G pump, Guardian Connect system, and GSR. For precision analysis between the MiniMed 640G insulin pump and Guardian Connect system, overall MARD between the MiniMed 640G insulin pump and Guardian Connect system values, using the MiniMed 640G insulin pump as the reference, was also calculated. The combined ±20%, ±30%, and ±40% agreement rates for YSI reference BG >80 mg/dL, or within ±20 mg/dL, ±30 mg/dL and ±40 mg/dL for YSI reference BG ≤80 mg/dL (hereafter referred to as %20/20, %30/30, and %40/40 agreement, respectively), between sensor-YSI glucose paired points across different YSI reference ranges, for each device, were also calculated. Sensor functional lifetime was determined from sensor data uploads through CareLink^™^ Clinical software and calculated as the time from the first sensor signal calibration pairing to the last SG value. In addition, subjects were administered a 7-point Likert scale questionnaire to assess their satisfaction with insertion, comfort, usability, and training. Analyses were performed using SAS version 9.4 software (SAS Institute, Inc., Cary, NC). Adverse events were collected throughout the training and study phases.

## Results

The mean ± SD age of the 88 eligible subjects (*n* = 46 males [52%], was 42.0 ± 19.1 years and their mean HbA_1c_ level was 7.9% ± 1.39%. Twenty-two subjects were 14–21 years of age and 66 were 22–75 years of age, 62 had type 1 diabetes, 10 had insulin-requiring type 2 diabetes, and 16 had noninsulin-requiring type 2 diabetes. Additional study subject characteristics, including mean body mass index, and prior insulin pump and CGM use are shown in Table 1.

**Table 1.** Characteristics of Eligible Study Subjects

*Characteristic*	*Subjects (*N* = 88)*
Female, *N* (%)	42 (47.7)
Male, *N* (%)	46 (52.3)
Age, mean ± SD, years	42.0 ± 19.08
Weight, mean ± SD, kg	83.5 ± 24.24
BMI, mean ± SD, kg/m^2^	28.2 ± 7.17
Diabetes classification, *N* (%)	
Type 1	62 (70.5)
Type 2, requiring insulin	10 (11.4)
Type 2, not requiring insulin	16 (18.2)
Prior CGM experience, *N* (%)	
Yes	36 (40.9)
No	52 (59.1)
Prior insulin pump experience, *N* (%)	
Yes	47 (53.4)
No	41 (46.6)

BMI, body mass index; CGM, continuous glucose monitoring; SD, standard deviation.

### Sensor description

**FIG. 2.**
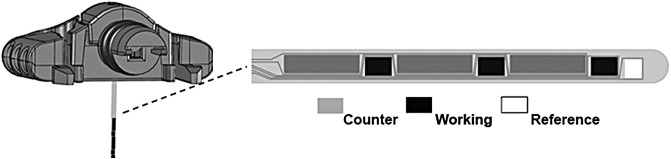
Guardian Sensor 3 sensor.

The Guardian Sensor 3 sensor (Fig. 2) is manufactured with similar materials, inserted into the skin at a 90-degree angle, and has the same implant depth (9.5 mm) as the currently marketed Enlite^™^ sensor. The sensor base profile dimensions (19.3 × 11.4 × 9.7 mm) and weight (2.91 g) are also similar to that of the Enlite sensor. However, the implanted sensor volume is reduced by ∼80%, because the tube encasing the sensor has been removed in the Guardian Sensor 3 sensor design. Substantial design changes significantly improving the sensor performance, reliability, and usability include the separation and distribution of the working and counter electrode across the implanted sensor surface (see Fig. 2), the focused application of enzyme over only the working electrode, and the optimization of sensor chemistry for accuracy and longevity. Guardian Sensor 3 sensor algorithm enhancements have focused on improving accuracy and reliability. The algorithm uses electrochemical impedance spectroscopy (EIS), which measures the complex impedance across different frequencies providing a proactive diagnostic of sensor health. Utilizing EIS proactively detects sensor faults, including sensor implant pullouts from the interstitial space and glucose sensitivity changes requiring recalibration.

### Sensor accuracy and precision

**FIG. 3.**
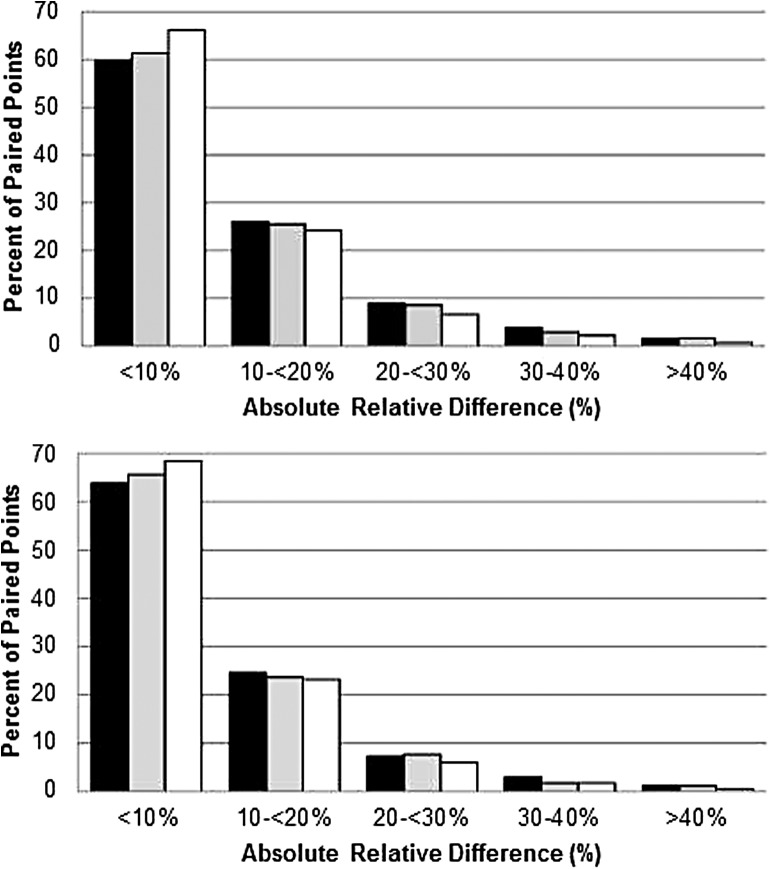
Distributions of overall ARD by device. The percentage of sensor glucose-YSI paired points associated with ranges (0% to >40%) of the overall ARD, according to each device, are shown when calibrating every 12 h **(Top)** and 3–4 times each day **(Bottom)**. The percent distribution of values when calibrating every 12 h and 3–4 times each day are listed in Table 3. Black: MiniMed 640G insulin pump, Gray: Guardian Connect system, White: Glucose Sensor Recorder. ARD, absolute relative difference; YSI, Yellow Springs Instrument.

**FIG. 4.**
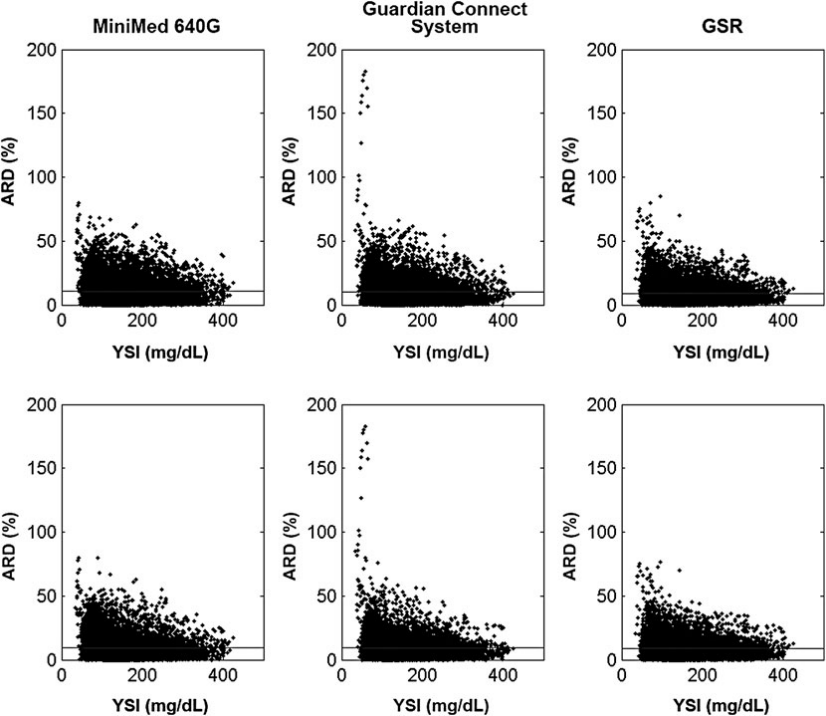
Overall ARD, as a function of YSI reference values, by device. The percent ARD distributions relative to YSI reference values (mg/dL) are shown, by device, when calibrating every 12 h **(Top)** and 3–4 times each day **(Bottom)**. The horizontal line across each graph indicates the mean. GSR, glucose sensor recorder.

Subjects were asked to undergo FST sessions on days 1, 3, and 7 of the study phase. Eighty-eight subjects completed day 1 testing, 87 completed day 3 testing, and 79 completed day 7 testing. The daily and overall MARD between SG and YSI reference measurements when calibrating every 12 h (i.e., minimum calibrations) and 3–4 times each day (i.e., one additional calibration) are shown in Table 2. When calibrating every 12 h, the overall MARD was 10.6% ± 9.6% (12,090 paired points) for abdomen sensors communicating with the MiniMed 640G insulin pump, 10.4% ± 10.4% (11,619 paired points) for sensors communicating with the Guardian Connect system, and 9.1% ± 8.3% (10,526 paired points) for sensors worn in the arm and connected to the GSR. When calibrating 3–4 times each day, the overall MARD was 9.6% ± 9.0% (11,664 paired points) for sensors communicating with the MiniMed 640G insulin pump, 9.4% ± 9.8% (10,937 paired points) for those communicating with the Guardian Connect system, and 8.7% ± 8.0% (10,771 paired points) for those worn in the arm and connected to the GSR. The percent distribution of overall MARD values by device, is listed in Table 3, and that of mean ARD is illustrated in Figure 3. The distribution of these values relative to YSI glucose reference is shown in Figure 4. The MARD, or precision, between MiniMed 640G insulin pump SG values and the Guardian Connect system SG values was 8.4% ± 9.93%.

**Table 2.** Guardian Sensor 3 Sensor Accuracy During Frequent Sample Testing, by Day and Device

			*Minimum calibrations^a^*		*One additional calibration^b^*	
*FST day*	*Device*	*Sensor location*	N	*MARD, %*	N	*MARD, %*
Day 1	MiniMed 640G pump	Abdomen	4294	13.0 ± 11.1	4136	11.7 ± 10.5
	Guardian Connect system	Abdomen	4013	12.4 ± 11.1	3728	11.2 ± 10.8
	GSR^c^	Arm	3390	10.8 ± 9.5	3591	10.3 ± 9.2
Day 3	MiniMed 640G pump	Abdomen	4533	8.9 ± 8.0	4378	8.3 ± 7.5
	Guardian Connect system	Abdomen	4294	8.7 ± 8.1	4125	8.2 ± 7.4
	GSR^c^	Arm	4243	8.1 ± 7.2	4198	7.8 ± 6.9
Day 7	MiniMed 640G pump	Abdomen	3263	9.5 ± 9.0	3150	8.7 ± 8.4
	Guardian Connect system	Abdomen	3312	10.1 ± 11.6	3084	8.9 ± 11.1
	GSR^c^	Arm	2893	8.5 ± 8.1	2982	8.1 ± 7.5
Overall	MiniMed 640G pump	Abdomen	12090	10.6 ± 9.6	11664	9.6 ± 9.0
	Guardian Connect system	Abdomen	11619	10.4 ± 10.4	10937	9.4 ± 9.8
	GSR^c^	Arm	10526	9.1 ± 8.3	10771	8.7 ± 8.0

Mean absolute relative difference is shown as Mean ± SD.

*N* = Number of sensor-YSI paired points.

^a^ Calibrating every 12 h.

^b^ Calibrating 3–4 times each day.

^c^ GSR data were obtained using the Guardian Link 3 transmitter calibration algorithm and calibration values from the MiniMed 640G insulin pump.

FST, frequent sample testing; MARD, Mean absolute relative difference.

**Table 3.** Percent Distribution of Overall Mean Absolute Relative Difference Values, by Device

	N	*0–<10%*	*10–<20%*	*20–<30%*	*30– <40%*	*>40%*
Minimum calibrations^a^						
MiniMed 640G pump	12090	59.7	26	8.9	3.9	1.5
Guardian Connect system	11619	61.3	25.5	8.6	2.9	1.6
GSR^b^	10526	66.2	24.2	6.6	2.2	0.7
One additional calibration^c^						
MiniMed 640G pump	11664	63.8	24.6	7.3	3	1.3
Guardian Connect system	10937	65.6	23.7	7.7	1.8	1.2
GSR^b^	10771	68.4	23.2	6	1.8	0.6

^a^ Calibrating every 12 h.

^b^ GSR data were obtained using the Guardian Link 3 transmitter calibration algorithm and calibration values from the MiniMed 640G insulin pump.

*N* = Number of sensor-YSI paired points.

^c^ Calibrating 3–4 times each day.

The overall %20/20, %30/30, and %40/40 agreements between SG and YSI paired points for YSI values in the low (≤70 mg/dL), normal (>70–180 mg/dL), and high (>180 mg/dL) glucose ranges, by device, are shown in Tables 4, 5, and 6, respectively, when calibrating every 12 h, and in Tables 7, 8, and 9, respectively, when calibrating 3–4 times each day. When calibrating every 12 h, the overall %20/20 agreement for sensors communicating with the MiniMed 640G insulin pump was 88.2% (10665/12090, within-agreement paired points/total paired points), the Guardian Connect system was 89.5% (10403/11619), and the GSR was 92.0% (9682/10526). The overall %30/30 agreement for sensors communicating with the MiniMed 640G insulin pump was 96.1% (11615/12090), the Guardian Connect system was 96.5% (11211/11619), and the GSR was 97.8% (10298/10526). The overall %40/40 agreement for sensors communicating with the MiniMed 640G insulin pump was 98.9% (11955/12090), the Guardian Connect system was 98.8% (11481/11619), and the GSR was 99.6% (10486/10526). When calibrating 3–4 times each day, the overall %20/20 agreement for sensors communicating with the MiniMed 640G insulin pump was 90.7% (10576/11664), the Guardian Connect system was 91.8% (10039/10937), and the GSR was 93.1% (10029/10771). The overall %30/30 agreement for sensors communicating with the MiniMed 640G insulin pump was 96.9% (11305/11664), the Guardian Connect system was 97.9% (10705/10937), and the GSR was 98.3% (10591/10771). The overall %40/40 agreement for sensors communicating with the MiniMed 640G insulin pump was 99.1% (11564/11664), the Guardian Connect system was 99.2% (10848/10937), and the GSR was 99.7% (10737/10771).

**Table 4.** Guardian Sensor 3 Sensor-Yellow Springs Instrument Paired %20/20 Agreement by Device, When Calibrating Every 12 Hours

*YSI reference range*	*%20/20 agreement (MiniMed 640G pump) Abdomen*	*%20/20 agreement (Guardian Connect system) Abdomen*	*%20/20 agreement (GSR*^a^) *Arm*
≤70 mg/dL^b^	92.5 (1056/1142)	93.2 (1065/1143)	93.1 (977/1049)
>70–180 mg/dL	87.0 (6242/7173)	88.3 (6098/6903)	91.3 (5738/6282)
>180 mg/dL	89.2 (3367/3775)	90.7 (3240/3573)	92.9 (2967/3195)
Overall	88.2 (10665/12090)	89.5 (10403/11619)	92.0 (9682/10526)

^a^ GSR data were obtained using the Guardian Link 3 transmitter calibration algorithm and calibration values from the MiniMed 640G insulin pump.

^b^ For reference blood glucose ≤80 mg/dL, agreement was based on ±20 mg/dL (Within-agreement paired points/total paired points).

YSI, Yellow Springs Instrument.

**Table 5.** Guardian Sensor 3 Sensor-Yellow Springs Instrument Paired %30/30 Agreement by Device, When Calibrating Every 12 Hours

*YSI reference range*	*%30/30 agreement (MiniMed 640G pump) Abdomen*	*%30/30 agreement (Guardian Connect system) Abdomen*	*%30/30 agreement (GSR*^a^) *Arm*
≤70 mg/dL^b^	99.3 (1134/1142)	98.3 (1124/1143)	99.0 (1038/1049)
>70–180 mg/dL	95.5 (6847/7173)	95.8 (6614/6903)	97.5 (6126/6282)
>180 mg/dL	96.3 (3634/3775)	97.2 (3473/3573)	98.1 (3134/3195)
Overall	96.1 (11615/12090)	96.5 (11211/11619)	97.8 (10298/10526)

^a^ GSR data were obtained using the Guardian Link 3 transmitter calibration algorithm and calibration values from the MiniMed 640G insulin pump.

^b^ For reference blood glucose ≤80 mg/dL, agreement was based on ±30 mg/dL (Within-agreement paired points/total paired points).

**Table 6.** Guardian Sensor 3 Sensor-Yellow Springs Instrument Paired %40/40 Agreement by Device, When Calibrating Every 12 Hours

*YSI reference range*	*%40/40 agreement (MiniMed 640G pump) Abdomen*	*%40/40 agreement (Guardian Connect system) Abdomen*	*%40/40 agreement (GSR*^a^) *Arm*
≤70 mg/dL^b^	99.8 (1140/1142)	98.9 (1130/1143)	99.7 (1046/1049)
>70–180 mg/dL	98.7 (7077/7173)	98.5 (6798/6903)	99.5 (6248/6282)
>180 mg/dL	99.0 (3738/3775)	99.4 (3553/3573)	99.9 (3192/3195)
Overall	98.9 (11955/12090)	98.8 (11481/11619)	99.6 (10486/10526)

^a^ GSR data were obtained using the Guardian Link 3 transmitter calibration algorithm and calibration values from the MiniMed 640G insulin pump.

^b^ For reference blood glucose ≤80 mg/dL, agreement was based on ±40 mg/dL (Within-agreement paired points/total paired points).

**Table 7.** Guardian Sensor 3 Sensor-Yellow Springs Instrument Paired %20/20 Agreement by Device, When Calibrating Three to Four Times Each Day

*YSI reference range*	*%20/20 agreement (MiniMed 640G pump) Abdomen*	*%20/20 agreement (Guardian Connect system) Abdomen*	*%20/20 agreement (GSR*^a^) *Arm*
≤70 mg/dL^b^	92.8 (1012/1090)	93.3 (992/1063)	93.8 (1004/1070)
>70–180 mg/dL	88.9 (6213/6990)	90.2 (5875/6516)	92.3 (5953/6453)
>180 mg/dL	93.5 (3351/3584)	94.5 (3172/3358)	94.6 (3072/3248)
Overall	90.7 (10576/11664)	91.8 (10039/10937)	93.1 (10029/10771)

^a^ GSR data were obtained using the Guardian Link 3 transmitter calibration algorithm and calibration values from the MiniMed 640G insulin pump.

^b^ For reference blood glucose ≤80 mg/dL, agreement was based on ±20 mg/dL (Within-agreement paired points/total paired points).

**Table 8.** Guardian Sensor 3 Sensor-Yellow Springs Instrument Paired %30/30 Agreement by Device, When Calibrating Three to Four Times Each Day

*YSI reference range*	*%30/30 agreement (MiniMed 640G pump) Abdomen*	*%30/30 agreement (Guardian Connect system) Abdomen*	*%30/30 agreement (GSR*^a^) *Arm*
≤70 mg/dL^b^	99.4 (1083/1090)	98.2 (1044/1063)	99.0 (1059/1070)
>70–180 mg/dL	96.2 (6725/6990)	97.3 (6338/6516)	97.9 (6316/6453)
>180 mg/dL	97.6 (3497/3584)	99.0 (3323/3358)	99.0 (3216/3248)
Overall	96.9 (11305/11664)	97.9 (10705/10937)	98.3 (10591/10771)

^a^ GSR data were obtained using the Guardian Link 3 transmitter calibration algorithm and calibration values from the MiniMed 640G insulin pump.

^b^ For reference blood glucose ≤80 mg/dL, agreement was based on ±30 mg/dL (Within-agreement paired points/total paired points).

**Table 9.** Guardian Sensor 3 Sensor-Yellow Springs Instrument Paired %40/40 Agreement by Device, When Calibrating Three to Four Times Each Day

*YSI Reference Range*	*%40/40 Agreement (MiniMed 640G Pump) Abdomen*	*%40/40 Agreement (Guardian Connect System) Abdomen*	*%40/40 Agreement (GSR*^a^) *Arm*
≤70 mg/dL^b^	100.0 (1090/1090)	98.8 (1050/1063)	99.7 (1067/1070)
>70–180 mg/dL	98.9 (6913/6990)	99.0 (6449/6516)	99.5 (6423/6453)
>180 mg/dL	99.4 (3561/3584)	99.7 (3349/3358)	100.0 (3247/3248)
Overall	99.1 (11564/11664)	99.2 (10848/10937)	99.7 (10737/10771)

^a^ GSR data were obtained using the Guardian Link 3 transmitter calibration algorithm and calibration values from the MiniMed 640G insulin pump.

^b^ For reference blood glucose ≤80 mg/dL, agreement was based on ±40 mg/dL.

(Within-agreement paired points/total paired points).

Summaries of the consensus error grid and Clarke error grid analyses during FST, when calibrating every 12 h, are shown in Tables 10 and 11. The summaries, when calibrating 3–4 times each day, are shown in Tables 12 and 13. For all devices, the percentage of aggregated evaluation points observed in the clinically accurate zones A + B for both metrics ranged from 99.1% to 99.9%, when calibrating every 12 h, and 99.2%–99.9%, when calibrating 3–4 times each day. For each device, <1% of evaluation points was observed in Zone E or D, for both metrics.

**Table 10.** Consensus Error Grid Analyses of Sensors by Device, When Calibrating Every 12 Hours

	*MiniMed 640G pump*		*Guardian Connect system*		*GSR*^a^	
	*Abdomen*		*Abdomen*		*Arm*	
	N	*%*	N	*%*	N	*%*
Zone A + B	12052	99.9	11577	99.8	10499	99.9
Zone A	10581	87.7	10362	89.4	9652	91.8
Zone B	1471	12.2	1215	10.5	847	8.1
Zone C	11	0.1	12	0.1	10	0.1
Zone D	0	0	6	0.1	0	0
Zone E	0	0	0	0	0	0

Values are from overall ranges (40–400 mg/dL).

^a^ GSR data were obtained using the Guardian Link 3 transmitter calibration algorithm and calibration values from the MiniMed 640G insulin pump.

*N* = Number of sensor-YSI paired points.

**Table 11.** Clarke Error Grid Analyses of Sensors by Device, When Calibrating Every 12 Hours

	*MiniMed 640G pump*		*Guardian Connect system*		*GSR*^a^	
	*Abdomen*		*Abdomen*		*Arm*	
	N	*%*	N	*%*	N	*%*
Zone A + B	11951	99.1	11496	99.1	10420	99.2
Zone A	10572	87.6	10292	88.8	9619	91.5
Zone B	1379	11.4	1204	10.4	801	7.6
Zone C	0	0.0	0	0.0	0	0.0
Zone D	111	0.9	99	0.9	89	0.8
Zone E	1	0.0	0	0.0	0	0.0

Values are from overall ranges (40–400 mg/dL).

^a^ GSR data were obtained using the Guardian Link 3 transmitter calibration algorithm and calibration values from the MiniMed 640G insulin pump.

*N* = Number of sensor-YSI paired points.

**Table 12.** Consensus Error Grid Analyses of Sensors by Device, When Calibrating Three to Four Times Each Day

	*MiniMed 640G pump*		*Guardian Connect system*		*GSR*^a^	
	*Abdomen*		*Abdomen*		*Arm*	
	N	*%*	N	*%*	N	*%*
Zone A + B	11631	99.9	10897	99.8	10745	99.9
Zone A	10504	90.3	10026	91.9	10001	93.0
Zone B	1127	9.7	871	8.0	744	6.9
Zone C	7	0.1	12	0.1	9	0.1
Zone D	0	0.0	6	0.1	0	0.0
Zone E	0	0.0	0	0.0	0	0.0

Values are from overall ranges (40–400 mg/dL).

^a^ GSR data were obtained using the Guardian Link 3 transmitter calibration algorithm and calibration values from the MiniMed 640G insulin pump.

*N* = Number of sensor-YSI paired points.

**Table 13.** Clarke Error Grid Analyses of Sensors by Device, When Calibrating Three to Four Times Each Day

	*MiniMed 640G pump*		*Guardian Connect system*		*GSR*^a^	
	*Abdomen*		*Abdomen*		*Arm*	
	N	*%*	N	*%*	N	*%*
Zone A + B	11541	99.2	10833	99.2	10675	99.3
Zone A	10490	90.1	9938	91.0	9964	92.7
Zone B	1051	9.0	895	8.2	711	6.6
Zone C	0	0.0	0	0.0	0	0.0
Zone D	96	0.8	82	0.8	79	0.7
Zone E	1	0.0	0	0.0	0	0.0

Values are from overall ranges (40–400 mg/dL).

^a^ GSR data were obtained using the Guardian Link 3 transmitter calibration algorithm and calibration values from the MiniMed 640G insulin pump.

*N* = Number of sensor-YSI paired points.

### Sensor functional life

Of the 98 sensors communicating with the MiniMed 640G system and expected to last to the end of day 7, the mean functional life was 145.9 ± 39.3 h (95% confidence interval [CI], 138.0–153.8), with a median functional life of 167.9 h (interquartile range [IQR], 139.6–167.9). Of the 92 sensors communicating with the Guardian Connect system and expected to last to the end of day 7, the mean functional life was 146.1 ± 41.6 h (95% CI, 137.5–154.7), with a median functional life of 167.9 h (IQR, 142.1–168.4). Of the 80 sensors inserted in the arm and connected to the GSR and expected to last to the end of day 7, the mean functional life was 147.6 ± 40.4 h (95% CI, 138.6–156.6), with a median functional life of 167.9 h (IQR, 147.3–167.9). While the duration of mean functional life was based on sensor signal communication with device transmitters, the specific information regarding removal or dislodgment of sensors was not captured.

### Overall safety

There were five adverse events during the study, none of which was serious or device related. These included one each of gastroenteritis, worsening of benign prostatic hypertrophy, rash at the site of IV access, upper respiratory tract infection, and blistering from skin tac used under tape. A total of 704 skin assessments of the sensor insertion sites were conducted on 88 subjects. Most skin assessments were related to redness at the insertion site or in the adhesive area, and were considered mild in nature.

### Overall satisfaction

Experiences with the sensor were generally favorable and demonstrated positive acceptance of the device. Regarding sensor insertion, comfort, usability, and training statements, all subjects reported median scores of 6 or 7 on a 7-point Likert scale, where seven reflects strong agreement with the statement. Examples of these statements included: “Sensor insertion was no more painful than a finger stick,” “The sensor insertion device was easy to use,” and “The training for inserting the sensor was effective.” Two questions involving instruction materials and 24-h Help Line assistance elicited median scores of 4 (neutral) and included: “I referred to the User Guide(s) often to troubleshoot” and “I would rather call the 24-h Help Line than read the User Guide, if I needed assistance.” A median score of 1 (strongly disagree) was observed for one statement: “I had to contact the 24-h Help Line often to troubleshoot.”

## Discussion

Commercially available RT-CGM systems can differ in visual display options; calibration requirements; and sensor specifications, electrochemistry, accuracy, reliability, and functional life. Known benefits of CGM include reduced HbA_1c_,^1–3,5–7^ glucose variability,^4,7,8^ and hypoglycemia,^9,10^ in addition to improved treatment satisfaction.^11,12^ Important end-user benefits of RT-CGM, when compared with point-in-time SMBG, have included the means to visualize current and trending glucose values that approximate blood glucose, and the ability to respond to those values in real-time. Tantamount to these benefits are the numerical accuracy (e.g., MARD relative to a standard venous reference) and clinical accuracy (e.g., Clarke Error Grid^19,20^ or Consensus^15^ Error Grid analysis); accuracy assessment metrics often used to quantify sensor accuracy and performance with respect to clinical decision making, respectively. Previous studies of earlier-generation Medtronic sensors reported an overall MARD of 13.9% (6404 sensor-YSI paired points), with 99.1% of evaluation points within the A + B zones of the Consensus Error Grid analysis, with the MiniMed Veo™ system algorithm^16^; and an overall MARD of 13.6% (7415 sensor-YSI paired points), with 98.8% of evaluation points within the A + B zones of the Consensus Error Grid analysis, with the MiniMed Revel™ system algorithm.^17^ Another study comparing sensor accuracy between two different generations of sensors (Dexcom G4^®^ PLATINUM and Dexcom SEVEN^®^)^21^ reported an overall MARD, relative to YSI, across multiple days of sensor wear of 13% and 16%, respectively.

The Guardian Sensor 3 sensor is a component of the MiniMed 670G HCL system, which was approved by the FDA in September 2016. The pivotal trial of the MiniMed 670G HCL system showed that the sensor allowed for the safe delivery of basal insulin, as per the HCL algorithm, in 124 subjects for 3 months, during which time there was no severe hypoglycemia or DKA in over 12,000 days of patient use.^19^ During a 6-day/5-night hotel stay conducted during the pivotal trial, SG values were compared with venous samples using the i-STAT^®^ (Abbott Laboratories, Abbott Park, IL) as reference, during a 24-h period. The overall MARD was 10.3%, for 3710 paired points evaluated on 7 days of sensor wear during the 3-month study, demonstrating that the sensor functions well in real-life use, as part of an HCL system.

For years, the performance and reliability of CGM technology have been deemed critical in the development of closed-loop insulin delivery systems.^20,22^ The current study demonstrated that the Medtronic Guardian Sensor 3 sensor, when inserted in the abdomen, has improved accuracy as determined by the within-percent agreement rates with YSI reference values and overall MARDs of 10.6% (when calibrating every 12 h) and 9.6% (when calibrating 3–4 times each day), for a duration of wear extended from 6 to 7 days. Even better accuracy (9.1% when calibrating every 12 h and 8.7% when calibrating 3–4 times each day) was observed for the sensor when located in the arm and connected to the GSR. While optimal sensor placement can be an important topic, location-specific influences on sensor signal stability or sensor longevity have not been studied. There are very few publications regarding glucose sensors located in the arm or forearm. One previous sensor accuracy study by another group reported a lower MARD for sensors located in the arm (12.6%), relative to the abdomen (13.1%).^23^ An earlier study investigating a prototype viscometric affinity glucose sensor^24^ reported a lower, but not significantly different, range of mean absolute relative error for sensors located in the arm (10.2%–13.7%) compared with that for sensors located in the abdomen (12.0%–16.6%). While the duration (5 days and 8 h) of sensor accuracy investigation for each of the aforementioned studies differed, neither postulated as to what might underlie accuracy differences between sensors placed in the different locations. There may be fewer mechanical stresses on sensors located in the arm, compared with the abdomen, which potentially influence accuracy. While the current study was not specifically designed to compare sensor performance across display systems or between sensor locations, advantages inherent to sensor location could play a role in sensor accuracy.

Limitations of this study include the fact that subjects were encouraged to calibrate the system at least four times per day (before meals and before going to bed) during home use, which exceeds the minimum system requirement of calibration at least every 12 h following the second calibration after sensor insertion. Although four calibrations per day (mean calibrations during the study were 3.7 ± 1.5 times/day) should be representative of real-life use of CGM systems, as this corresponds to system calibration before meals and before bedtime, the performance of the system when calibrated less frequently was not assessed. Another study limitation was that subjects were asked to wear three sensors and use two different systems simultaneously, whereas only one sensor and one system would typically be used during real-life use of a CGM technology. While this may have significantly increased use burden and resulted in a lower satisfaction rating for ease of use, questionnaire responses related to ease of use were very favorable. The sensor was well tolerated with none of the adverse events reported during the study being related to the devices or systems used. All results related to the examinations of the sensor insertion sites, after sensor removal, were mild in nature and typical of those anticipated for transcutaneous glucose sensors. These findings are favorable, especially in a cohort where over 50% of subjects completing the study phase had no prior CGM experience.

## Conclusions

The Guardian Sensor 3 glucose sensor was accurate and precise in the hypoglycemic, euglycemic, and hyperglycemic ranges when located in the abdomen and paired with either the MiniMed 640G pump or the Guardian Connect system, or when located in the arm and connected to a Glucose Sensor Recorder. These findings, and recently reported outcomes from the Medtronic HCL system pivotal trial,^18,19^ support the use of the sensor for standalone, open-loop, HCL, and closed-loop systems.

## Product Disclosure

The Medtronic MiniMed 640G system and Guardian Connect system are not available for commercial use in the United States.
